# Hospital Meetings

**Published:** 1893-09-09

**Authors:** 


### HOSPITAL MEETINGS, &C.

### THE LONDON HOSPITAL AND THE *PALL MALL GAZETTE*.

The Governors and the Allegations.

At the height of the holiday season, a sederunt of seventy-six at such a meeting as the Quarterly Court of the Governors of the London Hospital is surely worthy of remark. This was the number of ladies and gentlemen who made the Board-room of the London Hospital uncomfortably close on Wednesday afternoon; and it is not too much for one who was present at the meeting to say that this large attendance is to be attributed to the loyalty of the great body of Governors to the hospital, to their interest in its welfare, and in the great work that it is doing.

Mr. J. H. Buxton, the Treasurer of the Hospital, presided, and he was supported by Mr. John H. Hale, the Chairman.

The business that occupied the entire sitting came up on the report of the House Committee. That report set forth that the attacks which had been made on the Hospital in the *Pall Mall Gazette* had been under the consideration of the Committee; that a meeting had been held with the staff, at which the letter signed by the Chairman and others which had appeared in the *Pall Mall Gazette* had been drawn up; and that letters (which are reproduced in another column) had been addressed to the House Committee—one signed by 263 members of the present nursing staff, another by 241 past workers in the hospital protesting against the "imputations on the efficiency of the hospital" which had appeared in the *Pall Mall Gazette*. The recommendation of the Committee was in the following terms:—

"In view, therefore, of these strong refutations of such criticisms as have been made, the Committee have unanimously decided that no further action of any sort is advisable. They earnestly hope that the governors will co-operate in convincing the public of the little value to be attached to the articles that have recently appeared in the *Pall Mall Gazette*."

The Chairman (Mr. Buxton) moved the adoption of the report. Referring to the letters that had appeared in the *Pall Mall Gazette*, he said they were remarkable for their flinging of dirt without any regard to truth and without any knowledge of the work in the London Hospital. All the letters, with the honourable exception of that from Mrs. Hunter, had been anonymous. He wondered if the governors really knew what improvements had been made in the London Hospital during the past ten years. The place now wore an utterly different aspect. The policy pursued had been one of steady progressive reform. (Applause.) That hospital had led the way among the hospitals of this country in regard to internal reforms. The writers of the letters had made complaints as to the nurses' food. The fact was that the nurses were quite satisfied with their food. It is good, appetising, and with constant change. Moreover, it is cooked at their own kitchen, and ordered by nurses for the nurses. Members of the committee had sometimes paid a surprise visit to the nurses' dinner, and those who had done so had found everything in a most satisfactory condition. So far from suffering on account of the food they got, the nurses who came to that hospital as a rule got healthier. The truth was that the arrangement with reference to the supper meal was that nurses were allowed to suit their own liking and tastes. It had been said that the work of the nurses was incessant. How did that charge square with the fact that in addition to the dinner-time every nurse had at least two hours off every day? And this off-time, be it noted, was given not in the evening but during the best hours of the day. Further, in regard to the amount of work, there were at the London Hospital less than three patients per nurse—a lower figure than any other metropolitan hospital could show. An attempt had been made to dictate to them as to the time that the Matron should spend in the wards. Did those who spoke in this way know of the amount of work of all kinds that Miss Lückes had to do? Those who had had occasion to consult Miss Lückes would agree with him that "marvellous" was not too strong a word to apply to the knowledge which their Matron had of every detail of the work of the hospital, and of every individual on the nursing staff. (Applause.) Moreover, they had an Assistant Matron whose constant duty it was to look after the wards. Miss Lückes, who had been so bitterly attacked, and whom some of them disliked so much, regarded it as her first duty to makes the nurses comfortable, and after that to give them a thorough and complete training. (Applause.) It had been said that there was waste in the hospital. Yes, a certain amount of waste was unavoidable in an institution such as theirs. But the waste there could not be much when the cost per patient per day was so low. The sisters of the wards were most careful not to order for the patients under their care more than was probably needed, but the diets were ordered by the doctors. Mr. Buxton then referred to the letter addressed to the House Committee by 263 members of the present nursing staff. He wished to emphasize that this letter had been drawn up by the nurses themselves without the knowledge of any officer or any help but their own, and that it had been voluntarily signed by 263 out of the 275 who could sign. Having read the letter, Mr. Buxton said that the accusers of the hospital had been doing all they could for three years to stir up insubordination in that place. The success of these efforts was measured by the letter which he had read. (Applause.) The Chairman also read the letter from 241 past workers at the London Hospital which is reproduced in another page. These two letters, he said, showed what those who were and had been workers in the London Hospital thought of the charges which had been made. He could also tell them that the members of their medical staff—and he was very glad to see Sir Andrew Clark there that day—(applause)—felt very warmly that those malicious attacks must be answered. Let it not be said that the Committee did not wish for criticism and advice; but let those who wished to criticise or advise come to the Committee in a straightforward, honest way and say what they had to say. It was much easier to do harm than to keep things right. (Applause.) He moved the adoption of the report.

Mr. J. Hale (Chairman of the hospital) seconded. He heartily endorsed all that had been said with reference to the despicable attacks that had been made. He thought the time had now arrived when the governors must put their foot down and say they did not believe these reports. (Applause.) The endeavour of those who made these attacks was to injure the hospital as much as possible; and they thought they had chosen an auspicious time when they were endeavouring to increase their maintenance fund. He was glad, however, to say that the attacks had not been successful, as their maintenance fund, including annual subscriptions, now stood at £50,000. (Applause.)

Mrs. Hunter said that she wished at the outset to make it clear that those who had been instrumental in bringing evidence with regard to the London Hospital before the Lords' Commission had nothing to do with this attack in the *Pall Mall Gazette*. She held no brief for the *Pall Mall Gazette*. The new charges were similar to those which had been brought before the Lords' Committee. But she had to acknowledge that their House Committee had done a great deal since the time of that inquiry. Her (Mrs. Hunter's) regret was that they had not gone far enough. The sum expended on the food of the nurses at the hospital had increased every year since the time of the Lords' Committee. There had been much improvement in the food generally. The mortality among the nurses had decreased. The young nurses were not now put in charge of serious cases. She would urge on the committee the undesirability of meeting criticisms with point blank denial. (The Chairman: I did not do so.) In her opinion, said Mrs. Hunter, the house committee were imperfectly acquainted with the facts. In the article in The Hospital of August 5th under the head of "Statements that are True," it was admitted that the sister who had charge of the erysipelas ward was also sister for the isolation ward. What did they think of a committee which allowed a state of matters, thus condemned by its champion, to go on? (Laughter.) Mrs. Hunter urged that there should be a sub-committee to look after the nursing, and on this committee there should be women. (Laughter and applause.) She thought that the present House Committee would do well to make a few more changes.

The Rev. Mr. Wainwright (St. Peter's) said that he had been intimately acquainted with the hospital for twenty-one years. During all that time he had heard nothing but gratitude and unqualified praise on the part of those who were patients. (Applause.) He had told his people in church of the charges that had been made against the hospital, and the result was that they were resolved to hold a special collection for the London Hospital. (Applause.)

Mr. Hall said that he had not always agreed with the House Committee; but he felt it his duty to say that there was no institution in London which was more highly spoken of than the London Hospital.

Dr. Bedford Fenwick said the *Pall Mall Gazette* had put forward a challenge, and he thought it should be met. Although he should stand alone, he would move that the House Committee be asked to call upon the Editor of the *Pall Mall Gazette* either to prove his charges or withdraw them. It had been stated that nurses went from the erysipelas ward to nurse children who had undergone tracheotomy. If that were so, it was a most reckless disregard of ordinary precautions.

Mr. J. Hale: The nurses do not go from the one ward to the other, but the sister does, and that is allowed by the doctors.

Dr. Bedford Fenwick: It is most extraordinary. ("Oh, Oh!") He then moved that the committee call on the *Pall Mall Gazette* either to prove its words, or withdraw and apologise; and that an inquiry be held into the whole matter.

The motion was not seconded.

Sir Andrew Clark, who was received with cheers, said he had been for forty years on the staff of that hospital. He was very much afraid that neither hospitals nor men were born to perfection. (Hear, hear.) But he thought they should strive after perfection as much as they could. If that hospital had been doing so it was the duty of its friends to give it what help they could in a loving spirit. Any action that would interfere with the great work that the London Hospital was doing was an immoral action. (Applause.) After forty years' experience of it he could say that he knew of no hospital which in medical, nursing, and domestic management exceeded in rightness of work the London Hospital. (Applause.) He attributed no motives, but as a man of the world he felt there was a savour of malice in these charges. There was no just ground for the attacks. The effect of Dr. Bedford Fenwick's proposal that they should submit to a new inquiry would be that the hospital would be thrown into a confusion, and at the end of the inquiry they would be no better than they were that day. In declining the challenge the committee would be acting prudently and wisely. (Applause.)

Mr. Hunter having spoken briefly, the Chairman put the motion as to the adoption of the report, and it was carried *nemine contradicente*, and amid applause. A vote of thanks to the Chairman brought a sitting that had lasted over two hours to a close.

### THE MIDDLESEX HOSPITAL.

### Quarterly Court.

The quarterly court of the governors of this hospital was held in the board-room of the hospital on Thursday of last week, Mr. Robert Reid in the chair. The meeting was a gratifying one, inasmuch as several legacies were reported, but otherwise it presented no special feature. The report of the weekly board, read by Mr. Melhaldo, the secretary, bore out that it had been decided to reconstruct the operation theatre; and that a site for a convalescent home at Clacton-on-Sea, which seemed to be suitable in every way, had been purchased for £1,500. Appropriate reference was made in the report to the death of Mr. Foster, lecturer on chemistry in the school, who had been succeeded by Dr. Plympton. A report was made as to the work of renovating the buildings, which has necessitated the closing of the hospital for some time. It will be reopened on the 18th of this month. The sum collected in alms boxes outside the hospital during the quarter was stated to be £74 1s., the largest amount ever received from these boxes. The alms boxes within the hospital have yielded £19 14s.

### THE POPLAR HOSPITAL FOR ACCIDENTS.

### The Opening of the New Wing.

Nobody could be in the neighbourhood of Poplar Hospital on Monday afternoon without realising that something unusual was going on. Evidently the hospital was the centre of attraction. Poplar, young and old, lined the street in front of it, and the buildings rejoiced in a profusion of flags and banners with which they are not decked every day. But there was good reason for the crowds and for the tasteful display in the fact that on that afternoon the new wing to the hospital, which has been a-building for nearly two years, was to be formally opened. "A new wing" it is called in the card of invitation issued by the committee; it is really a new hospital. The facts are these: Poplar Hospital for Accidents has been in existence for over thirty-eight years. During that time it has done invaluable work in dealing with the cases of accidents that arise daily, almost hourly, at the docks and elsewhere in the neighbourhood. The nearest general hospital is the London, and it is two milos off. The Poplar Hospital not only relieved the strain upon the London, but it afforded hospital accommodation in the heart of a district where accidents were most frequent. The importance of nearness to an hospital in the case of a serious accident is so obvious that one need not dwell upon it. Nevertheless, the Poplar Hospital has had to do its work all these years in very unsatisfactory buildings. These were old, uncomfortable, and, latterly at any rate, insanitary. It was brought home to the members of the Hospital Committee that it would be absolutely necessary to obtain fresh quarters. Eventually a plan of reconstruction was adopted, under which the old building would be gutted and fitted up anew for the purposes of administration; and, adjoining the old, an entirely new building, erected with room for 60 beds, instead of 40 as in the old, and also for the out-patient department, which is very large. The committee were fortunate enough to enlist a good deal of sympathy with the project, and among others the Hon. Sydney Holland threw himself into the movement with genuine enthusiasm. Thanks to the exertions of friends, this scheme has now been carried out, in so far that on Monday the hospital, which had been closed for some weeks, was reopened, this time in new buildings. The old hospital has been abandoned to administration, for which it is being suitably fitted up. A complete little hospital, with three wards of 20 beds each, and a large out-patient department, has taken its place. Although the new building has been opened, not until the extensive alterations upon the old have been completed will everything be in working order. The first floor of the new wing, which is eventually to form a ward, has at present to be utilised for the accommodation of the staff and other kindred purposes; but even now, if our impression be correct, there are 40 beds available. The increased accommodation will enable the committee to make an important concession to the women of Poplar. In the old hospital there was no female ward. In the new, the ward of 20 beds on the third floor, named the John Coles Ward, will be set apart for women and children.

This new hospital has not been built without money, and to raise the £25,000 needed to complete the whole has been a matter of some anxiety to the committee. All things considered, they have been very successful. On Monday morning only £2,500 of the needed sum remained to be raised, and by Monday evening that sum had been reduced by over £1,200. The committee has, therefore, good reason to hope that "by the spring of 1894, when the grand opening of the whole building will take place," the entire amount will have been realised. One most gratifying feature of the movement for a better hospital has been the genuine interest which the working men of the district have evinced in the matter. To this tangible expression was given on Monday, not only in the large turn-out of working men and the trades procession that took place in honour of the occasion, but in the substantial subscriptions that were announced from the working men's friendly societies.

The opening ceremony took place at five o'clock in the ward on the second floor, which has been named after Sir Donald Currie. It was tastefully decorated and comfortably seated, and it was full to the door. The committee had secured the services of Lord and Lady Knutsford to perform the opening ceremony. There was a special appropriateness in this, inasmuch as the hospital has been befriended in most signal fashion by the Hon. Sydney Holland, who is the son of Lord and Lady Knutsford. The platform party included Lord and Lady Knutsford, the Hon. Sydney Holland, most of the members of the general committee of the hospital, and Mr. A. F. Hills (president), who took the chair.

The Chairman congratulated the audience upon the occasion for which they were met. In the whole of the new building there was not a corner of which they might not be proud. They welcomed Lord and Lady Knutsford not only because of their high position in the State, but also because they were the parents of their son, the Hon. Sydney Holland, of whose services to the hospital it would be impossible to speak too highly. (Applause.) Mr. Hills then gave an account of the means that had been used to raise money. He dwelt with special satisfaction on the help which had been given by the working classes. As in the past so in the future, the prosperity of that hospital would rest upon the gifts of the poor.

Dr. Corner, senior honorary surgeon, said that only those who had had experience of the old building could realise the advantages of the new. Having given some interesting particulars as to the history of the hospital, mentioning that from the first the working classes had contributed to its maintenance, he paid a special tribute to Mr. Sydney Holland.

Mr. Cole, Secretary of the Canning Town and Plaistow United Trade and Benefit Societies, intimated a subscription to the building fund from those societies of £85. (Cheers.)

Mr. J. MacNulty, working man representative on the Hospital Committee, spoke in very warm terms of the services of Mr. Sydney Holland, and paid a high tribute to Miss Vacher, the Matron, and the members of the nursing staff.

Lord Knutsford, who was then called upon, was received with cheers. He expressed the great pleasure that Lady Knutsford and he had in being present at the opening of a building which would not only be an ornament to the neighbourhood, but of incalculable benefit to men in the East-end, and not to men only, but to women also. For thirty-eight years women had been knocking at their door, and they had at last gained admission. They Londoners were very proud of London. One thing in which they took a special and just pride was their hospitals. Among the London hospitals he (Lord Knutsford) regarded that hospital at Poplar as one of the most interesting. It was situated near the Docks, where accidents were very frequent; it was in the very centre of the place where it was most wanted. Supported by thousands of working men, it was truly a working man's hospital. Lord Knutsford spoke in high terms of the nursing and surgical staff. He noted that, in spite of the disadvantages of the old hospital, during thirty-eight years, there had not been a single case of hospital fever. Last year 633 accidents had been treated in that hospital; of these only 33 had proved fatal. He wished to thank the committee for the loyal support they had given to his son. He was proud of the work of his son. (Applause.) He would not praise him to his face, but he would say that his son had every reason to be thankful that the help which he had received had enabled him to do so much.

Lieut.-Colonel Feneran, House Superintendant, read a list of those who had agreed to subscribe towards liquidating the debt of £2,500. The list showed that £1,233 had been promised, a result which was received with applause.

Dr. Brownfield moved a vote of thanks to Lord and Lady Knutsford and Mr. Dack, who intimated a subscription of 40 guineas from the working men of Poplar, seconded.

Lady Knutsford was then asked to declare the building open, and, in doing so, said that it was no mere form when she expressed her great interest in that hospital with which her son was so closely connected, and her deep sympathy with the work they were going to carry on there. In that room they knew that in the days to come there would be much suffering—fathers would lie disabled, and parents would witness the suffering of their children; but they could now rejoice in the relief that would be afforded to the sufferers through the skill of their medical men and the tender care of the good women who were the nurses. She hoped that God's blessing would rest on the buildings. Lady Knutsford then declared the new wing open.

The formal proceedings terminated at this point, but an invitation was extended to all to make an inspection of the buildings. Of this invitation a large number of ladies and gentlemen availed themselves. A little later there was a procession of trades, in which most of the trades of the district took part. The workmen marched to the hospital, and, having given hearty cheers for Mr. Sydney Holland and the new hospital, dispersed.

